# The tissue dependent interactions between p53 and Bcl-2 *in vivo*

**DOI:** 10.18632/oncotarget.5372

**Published:** 2015-10-06

**Authors:** Xin Li, Xiao Miao, Hongshen Wang, Zhixiang Xu, Bin Li

**Affiliations:** ^1^ Department of Dermatology, Yueyang Hospital of Integrated Traditional Chinese and Western Medicine, Shanghai University of Traditional Chinese Medicine, Shanghai, China, 200437; ^2^ Shanghai University of Traditional Chinese Medicine, Shanghai, China, 201203; ^3^ Division of Hematology/Oncology, Department of Medicine, University of Alabama at Birmingham School of Medicine, Birmingham, AL 35233, USA

**Keywords:** p53, Bcl-2, apoptosis, interaction, mice

## Abstract

To further investigate the role of *p53* in apoptosis *in vivo* and the interaction between *p53* and *Bcl-2* in the regulation of cellular apoptosis *in vivo*, we depleted *p53* in *Bcl-2*-null mice. We found that the interaction between *p53* and *Bcl-2* are tissue dependent. Specifically, loss of *p53* in *Bcl-2^−/−^* mice inhibits apoptotic induction in spleen and subsequently inhibits the *Bcl-2*-null-induced spleen atrophy. Furthermore, *p53* deficiency overcomes loss of melanocyte stem cell (MSC)-induced apoptosis and subsequently prevents hair graying in *Bcl-2-* null mice. In addition, *p53* deletion partly inhibits apoptosis in hair follicle keratinocytes, leading to the alleviation of hair growth delay in *Bcl-2-*null mice. However, *p53* absence in *Bcl-2*-null mice cannot restore other defects in *Bcl-2-*null mice, including retardation of growth, short ears and polycystic kidney disease.

## INTRODUCTION

The *p53* tumor suppressor activates a series of diverse antiproliferative responses to protect cells from different cellular stresses. One of the most important *p53* functions is its ability to activate apoptosis in a stage-, tissue- and stress-signal specific manner, and disruption of this process can promote tumor development [1]. As a hallmark of cancer, mutant *p53* was involved in resistance to apoptosis, genomic instability, aberrant cell cycle, invasion and metastasis, tumor microenvironment, and inflammation [2].

Two major apoptotic pathways mediate *p53*-stimulated apoptosis, including the B-cell leukemia lymphoma 2 (*Bcl-2)* family and the caspase cascade. Members of *Bcl-2* family include the ‘multidomain’ *Bcl-2* family member *Bax,* the ‘BH3-only’ members *Puma* [3], *Noxa* [4, 5], and *Bid* [6]. *Bcl-2* belongs to the *Bcl-2* family proteins, which is one of the most important regulators of the mitochondrial apoptosis pathway [7–9]. It has been proved that the severity and extensiveness of the phenotype of *Bcl-2* deficiency could be greatly affected by multiple genetic elements of the mice, resulting in tissue-specific modulations of the cell death program during development and cellular homeostasis [10]. Deficiencies of different *Bcl-2* family proteins in mice result in multiple phenotypes, some of which are lethal [11]. Specifically, *Bcl-2* deficiency in mice leads to various abnormalities, such as increased embryonic death, lymphocytopenia, hypopigmentation, polycystic kidney disease (PKD), distorted small intestine, abnormal skeletal development, reduced body weight, postnatal growth retardation and shortened life span [12–18]. These results indicate that the *Bcl-2* protein has pleiotropic effects on embryonic development and adult homeostasis in various tissues and cells *in vivo*.

The interaction between *p53* and *Bcl-2* has been intensely studied *in vitro*. Specifically, *p53* interacts with members of the *Bcl-2* family (such as *Bcl-2*, *Bcl-XL* and *Mcl1*) of apoptosis-regulatory proteins in the cytoplasm to trigger apoptosis and to inhibit autophagy [19, 20]. Furthermore, the functions of *Bcl-2* on apoptosis are suppressed by the *p53* proapoptotic protein [21]. Interestingly, several independent studies have shown that *p53* affects transcriptional activity of a partial *Bcl-2* promoter [22–24]. In addition, blocking *p53-Bcl-2* interaction decreases Granzyme B-induced *Bax* activation, cytochrome c release, and hence effector caspase inactivation, resulting in a reduced sensitivity of target cells to both Granzyme B and CTL/NK-mediated cell death [25]. However, it remains unknown the interaction between *p53* and *Bcl-2 in vivo*. To further characterize the potential interaction between *p53* and *Bcl-2 in vivo*, we depleted *p53* in *Bcl-2*-null mice. We found that the *Bcl-2* knockout phenotype was alleviated by *p53* protein deletion in a tissue specific manner. Loss of *p53* in *Bcl-2^−/−^* mice inhibited apoptosis induction in spleen and hair follicle melanocytes and keratinocytes, but not in kidney.

## RESULTS

### Loss of *p53* in *Bcl-2^−/−^* mice prevents spleen atrophy, but does not restore normal growth and ear length of the mice

Loss of *Bcl-2* itself induces striking abnormalities in mice. Although these animals appear normal at birth, they fail to thrive, develop short ears, and their thymus and spleen degenerate; later, their coats turn gray and the mice die within 4 to 16 weeks from renal failure due to polycystic kidney disease. Cell death is prominent in the affected tissues, and the lymphocytes are abnormally sensitive to diverse cytotoxic stimuli [12].

To eliminate any variability due to genetic background, we analyzed only *Bcl-2* on a C57BL/6 background [26]. The *Bcl-2*^−/−^ phenotype was proved more uniformly severe than reported in mixed backgrounds [27]. To determine the effect of *p53* loss on the *Bcl-2^−/−^* phenotypes *in vivo*, we generated mice double null for *Bcl-2* and *p53*. For mating, the *p53* status was maintained in the heterozygous state to avoid potential broad genomic consequences arising from *p53* deficiency.

**Figure 1:**
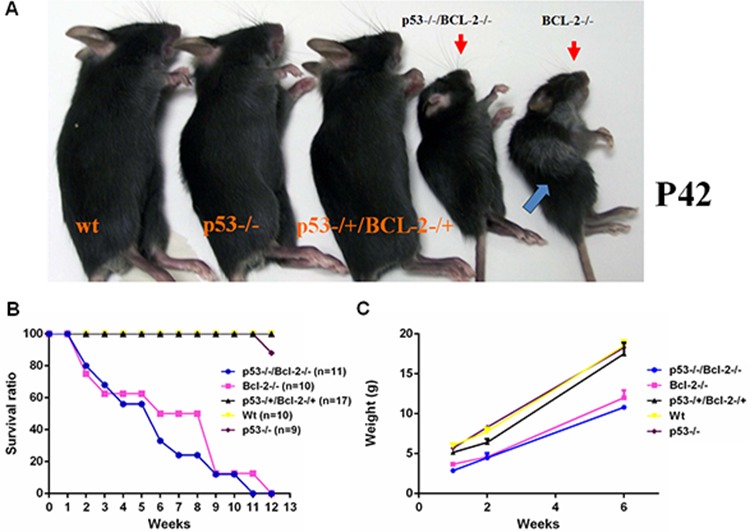
Loss of p53 prevents hair graying caused by Bcl-2 deficiency, but does not restore normal growth **A.** wt, *p53*^−/−^, *p53*^− /+^*/Bcl-2*^− /+^, *p53*^−/−^*/Bcl-2*^−/−^ and *Bcl-2*^−/−^ mice. Both *p53*^−/−^*/Bcl-2*^−/−^ and *Bcl-2*^−/−^ groups exhibited growth retardation with small body size and short ears. Hair graying is also usually observed in adult *Bcl-2*-null mice (arrow). **B.** Mean survival curve of mice of the indicated genotypes. The observed numbers were as shown. **C.** Mean body weight of mice of the indicated genotypes at different ages. Six mice were observed in each group.

**Figure 2:**
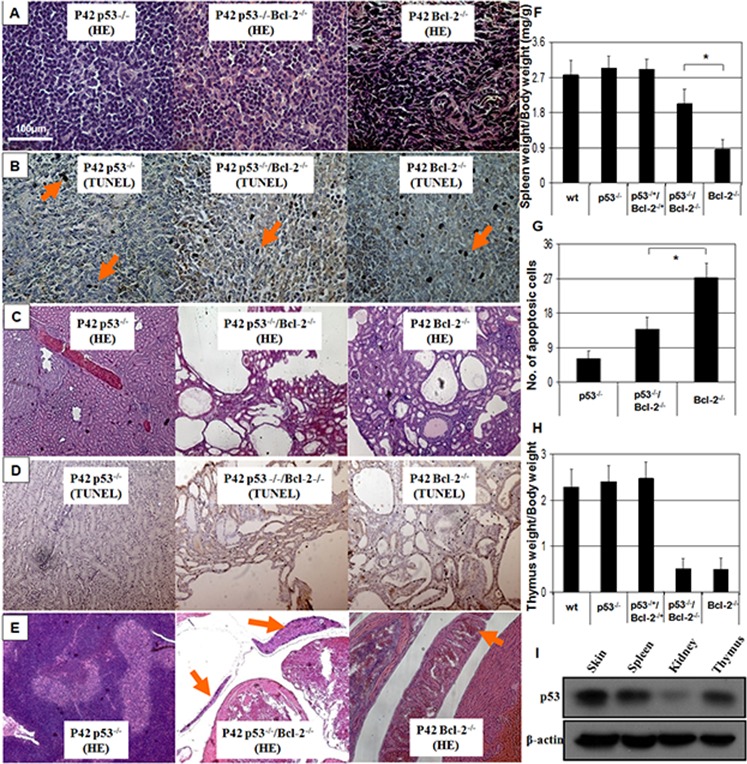
Spleen degeneration in Bcl-2^−/−^ mice is prevented by absence of p53 **A.** Hematoxylin and eosin staining of spleen sections ( × 100). Splenic degeneration was found in *Bcl*-*2*^−/−^mice and was rescued by *p53* deficiency in the double knockout. **B.** TUNEL staining for spleen sections showed hyper-apoptotic cells in *Bcl*-*2*^−/−^, which was prevented by loss of *p53*. **C.** Kidney sections ( × 100) show the abundant large thin-walled cysts in typical *Bcl*-*2*^−/−^ and *Bcl-2^−/−^*/p53*^−/−^* mice. **D.** TUNEL staining of kidney sections showed hyper-apoptotic cells both in *Bcl-2^−/−^* and *Bcl-2^−/−^*/p53*^−/−^* mice. **E.** Thymus degeneration was seen in the *Bcl*-*2*^−/−^ background with or without *p53* ( × 100). **F.** Spleen weights were evaluated at P42. Six mice were observed in each group. Asterisk indicates statistical significance (*P* < 0.01). **G.** Numbers of apoptotic cells in the spleen sections of *Bcl-2^−/−^* and *Bcl-2*^−/−^/*p53*^−/−^ mice were calculated. Asterisk indicates statistical significance (*P* < 0.01). **H.** Thymus weights were evaluated at P42 (*P* > 0.05). **I.** The *p53* protein expression in skin, kidney, spleen and thymus *in vivo*.

All *Bcl-2*^−/−^ mice that we produced had short ears, soon became runts (Figure 1A), and succumbed to polycystic kidney disease between the third and eighth week of life. Strikingly, all the double knockout mice (*n* = 17) exhibited growth retardation with short ears (Figure 1A), polycystic kidneys, thymus degeneration, low body weight (Figure 1C & Figure 2C, E, F and H) and died from 2 to 12 weeks old (Figure 1B). However, spleen degeneration was rescued (Figure 2A). We stained apoptotic cells in the spleen and kidney of adult mice in order to determine whether the interaction between *p53* and *Bcl-2* is tissue specific. In spleen, cell morphology is normal in the double-null mice (Figure 2A). Pertinently, apoptotic cells were especially abundant in the *Bcl-2*^−/−^ spleen compared to the double knockout mice (Figure 2B). In the kidney, TUNEL staining revealed similar numbers of positive cells in wild-type and *p53* and *p53*-null *Bcl-2*^−/−^ deficient mice (Figure 2D). Thus, loss of *p53* is strongly anti-apoptotic in spleen, but not in kidney. As a control, we detected the expression of *p53* protein in mouse skin, kidney, spleen and thymus *in vivo* and found that the expression level of *p53* protein is lower in kidney than in skin, spleen and thymus, all of which have same expression levels of *p53* protein (Figure 2I).

### Graying is prevented by the loss of *p53* in the *Bcl-2* deficient mouse

**Figure 3:**
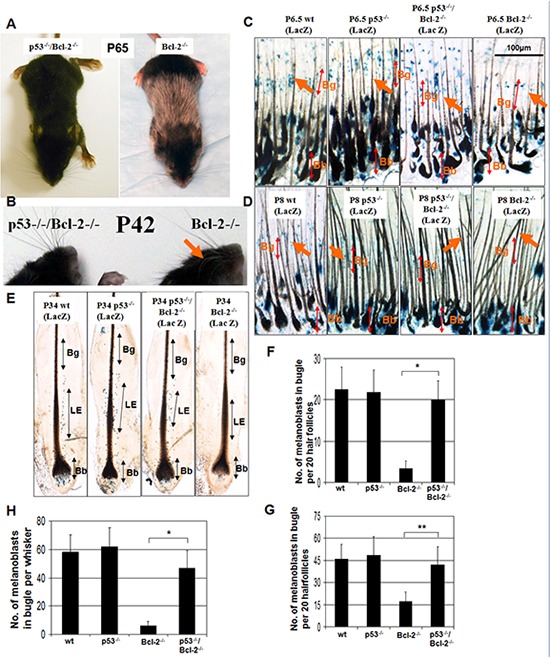
Graying elicited by Bcl-2 deficiency reflects p53-driven melanocyte death **A.** *Bcl*-*2*^−/−^, and *Bcl-2*^−/−^/*p53*^−/−^ C57BL/6 mice. Sixty-five day old *Bcl-2*^−/−^ mice turned gray from the second hair follicle cycle, whereas the coats of *Bcl*-*2*^−/−^/*p53*^−/−^ mice remained almost completely black although both groups exhibited growth retardation with small body size and short ears. **B.** Compare with *Bcl-2*^−/−^/*p53*^−/−^ mice, the whiskers of *Bcl*-*2*^−/−^ mice also turned gray (arrow). **C and D.** Distribution of *Dct-lacZ*^+^ melanoblasts (arrows) in the bulge region (Bg) (top double arrow) of pelage follicles at P6.5 and P8. Whereas bulb (Bb) melanocytes appear largely unchanged (bottom double arrow), bulge melanoblasts are unchanged in *Bcl-2^−/−^*/p53*^−/−^* follicles but lower in *Bcl2*^−/−^ follicles at P6.5 and lost at P8 compared with wt and *p53^−/−^* follicles. **F and G.** The frequency of *Dct-lacZ*^+^ melanoblasts per 20 follicles in the bulge region. Asterisks indicate statistical significance (*P < 0.01, **P < 0.05). **E.** Distribution of *Dct-lacZ*^+^ melanoblasts in the niche: lower enlargement (LE) (double arrow) of whisker hair follicles at P34. Compared to wt and *p53^−/−^* follicles and whiskers, LE melanoblasts were unchanged in *Bcl-2^−/−^*/p53*^−/−^* follicles but lower in *Bcl2*^−/−^ whiskers, and Bb melanocytes appear largely unchanged (bottom double arrow) in *Bcl-2^−/−^*/p53*^−/−^* follicles, but were lost in *Bcl-2^−/−^* whiskers. **H.** The quantity of *Dct-lacZ*^+^ melanoblasts per whisker in the LE. Asterisks indicate statistical significance (*P* < 0.01).

Hair graying is also usually observed in adult *Bcl-2*-null mice. To determine the potential impact of *p53* on the melanocytes in *Bcl-2*-null background, we observed the hair graying in *p53^−/−^*, Bcl-2*^−/−^* and double knockout mice at different ages. During the second hair follicle cycle at 5–7 weeks of age, *Bcl-2^−/−^* mice (*n* = 10) developed a gray coat as reported [12, 14, 26, 28], and removal of *p53* (*n* = 9) almost completely prevented the coat color change (Figure 3A). Loss of one allele of *p53* was not able to rescue hair graying in *Bcl-2* deficient mice (data not shown). The whiskers became gray starting at P20 in *Bcl-2^−/−^* mice and were rescued by loss of p53 as well (Figure 3B). We cannot conclude whether hair graying was delayed or prevented by loss of *p53* in *Bcl-2^−/−^* mice because double knockout mice all died by 12 weeks of age.

### Death of *Bcl2*^−/−^ melanocyte stem cells is rescued by the absence of *p53*

Hair follicles contain a well-demarcated structure for the stem-cell niche (within the lower permanent portion), whereas differentiated melanocytes reside in the hair bulb (at the base of the transient portion of the hair follicle) [26, 29, 30]. Although the graying of *Bcl-2*^−/−^ mice was originally ascribed to defective melanin synthesis [12], observations support the conclusion that it reflects the loss of melanocytes stem cells in the hair follicle [28]. Taking advantage of the spatial segregation of the stem versus differentiated cell compartments, we used melanocyte-targeted (*Dct*) *lacZ* transgenic mice [26, 29, 31, 32] with *Bcl-2*^−/−^, *p53^−/−^*, or both to examine the impact of aging on these melanocyte compartments.

At P6.5, hair follicle morphogenesis is almost complete. *Bcl-2*^−/−^ follicles contained melanoblasts in the bugle area (Figure 3C), whereas *Bcl-2^−/−^*/p53*^−/−^* follicles exhibited significantly more melanoblasts in the similar area (Figure 3C and 3F). In contrast, *Bcl-2*^−/−^ follicles at P8 showed nearly a complete loss of melanoblasts in the niche (bulge area), whereas melanoblasts still appeared in the niche of *Bcl-2^−/−^*/p53*^−/−^* follicles (Figure 3D and 3G). However, the number of melanocytes in the hair bulb did not show any differences between *Bcl-2^−/−^*/p53*^−/−^* and *Bcl2*^−/−^ mice in both time periods (data not shown). We also examined the numbers of melanocytes in the whiskers of *Bcl-2^−/−^* mice at P34. Melanoblasts were lost at this stage in the niche (lower enlargement area) and melanocytes were lost in the bulb as well in *Bcl-2^−/−^* follicles, whereas *Bcl-2^−/−^*/p53*^−/−^* follicles still contained melanoblasts in the niche and melanocytes in bulb (Figure 3E and 3G).

### Hair growth is delayed by hyper-apoptosis in *Bcl-2^−/−^* follicles, which is partly rescued by the loss of *p53*

**Figure 4:**
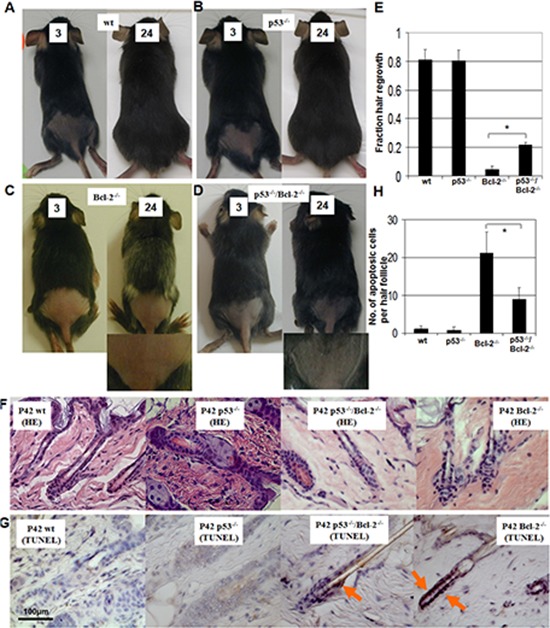
Hair regrowth in wild-type *(wt), p53^−/−^*, Bcl-2*^−/−^*/p53*^−/−^ and *Bcl-2*^−/−^* mice Hair regrowth assays were done in *wt* **A.** *p53*^−/−^ **B.** *Bcl-2^−/−^* **C.** and *Bcl-2^−/−^*/p53*^−/−^* **D.** mice. Mice were shaved the fraction of a 2-cm^2^ back area near the tail at 18 days old and observed every day for 24 days. The hair-growth assay was performed as described [34, 35]. Days after depilation (3 or 24 days) are indicated. An amplified picture is shown for *Bcl-2^−/−^* C. and *Bcl-2^−/−^*/p53*^−/−^* D. Each condition was done in triplicate per group. **E.** Mean hair growth is shown for *wt*, *p53*^−/−^, *Bcl-2^−/−^*, and Bcl-2*^−/−^*/p53*^−/−^* mice at P42. Asterisks indicate statistical significance (*P* < 0.01). **F.** Photomicrographs of histological sections of the hair follicles at P42. HE staining, 400 ×. **G.** TUNEL staining of hair follicle sections showed hyper-apoptotic cells in *Bcl*-*2*^−/−^ mice, which was partly prevented by the loss of *p53*. **H.** Numbers of hair follicle apoptotic cells (TUNEL-positive) in *Bcl-2^−/−^* and *Bcl-2*^−/−^*p53*^−/−^ mice were calculated compared to controls. Asterisk indicates statistical significance (*P* < 0.01).

A previous report demonstrated that *Bcl-2* protein is highly expressed in p53 null hair follicles. Furthermore, *p53* protein is involved in the control of murine hair follicle regression *in vivo* [33]. To identify the role of *Bcl-2* absence in hair growth and the interaction between *Bcl-2* and *p53* in hair growth, we assayed hair growth in *p53^−/−^*, Bcl-2*^−/−^* and double knockout mice. Hair follicles are constantly renewing, with alternating phases of growth (anagen), regression (catagen), and rest (telogen). The hair on the adult *Bcl-2^−/−^* mice (with or without *p53*) appeared to be sparser than that of their *Bcl-2*^+/+^ counterparts. Hair sparseness could be a function of the ratio of hair follicle cells in the anagen (growth) phase to those in the telogen (resting) phase. To test this, age-matched wild-type (*wt*), *p53*^−/−^, *Bcl-2^−/−^*/p53*^−/−^* and *Bcl-2^−/−^* mice (18 days old) were shaved in a 2-cm^2^ area on the back near the tail using an electric razor and then depilated. The subjective element in estimating the area is minimized by deficient regrowth as the first appearance of hair, and dividing the shaved area to be scored into eight equal portions with a transparent screen for hair-growth assay as described [34, 35]. A depilation-induced murine hair cycle is twenty-five days. In this period, the anagen phase is from day 1 to day 12, the catagen phase is from day 17 to day 18, and the telogen phase is day 0 or day 25 [36]. We measured hair regrowth one hair cycle (24 days) after depilation. Almost no hair growth was observed in *Bcl-2^−/−^* mice (Figure 4C and 4E). Hair growth declined linearly *Bcl-2^−/−^*/*p53^−/−^* mice (Figure 4D and 4E), whereas *wt or p53^−/−^* mice displayed robust hair growth (Figure 4A, 4B, 4E) and exhibited no hair cycle changes as reported [33]. Histology and apoptosis of hair follicles were observed on the age-matched *wt*, *p53*^−/−^, *Bcl-2^−/−^*/p53*^−/−^* and *Bcl-2^−/−^* mice. We couldn't find any marked defect in the hair follicles of all groups of mice (Figure 4F). As expected, *Bcl-2^−/−^* mice, compared to *wt* and *p53*^−/−^ mice, were characterized by the significantly increased (*P* < 0.01) number of TUNEL-positive cells and this increase was partly prevented by the loss of *p53* (Figure 4G and 4H). This suggests that deletion of *Bcl-2* induces apoptosis in follicles and retards the hair growth. This process can be partially prevented by loss of *p53*.

### Loss of *p53* inhibted the cell death induced by *Bcl-2* knockdown

**Figure 5:**
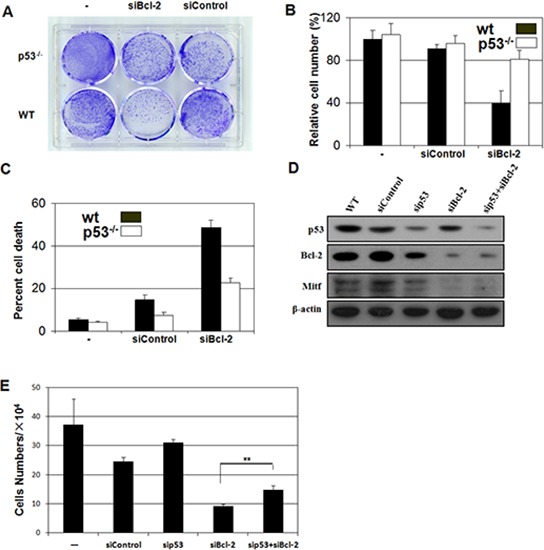
Loss of p53 inhibits the apoptosis induced by absence of Bcl-2 *in vivo* or Bcl-2 knockdown *in vitro* **A.** *p53^−/−^* melanocytes are relatively resistant to cell death driven by knockdown of *Bcl-2*. Mouse primary melanocytes were plated and transfected with control or siRNA as indicated. Cells were fixed and visualized by dye staining. **B.** *p53^−/−^* mouse primary melanocytes were refractory to proliferation after knockdown of *Bcl-2*. Equal numbers of mouse primary melanocytes were transfected with vector or siRNA as indicated. **C.** Wild-type, but not *p53^−/−^*, mouse primary melanocytes showed reduced survival in response to the si*Bcl-2*. Viability and apoptosis in mouse primary melanocytes was determined by flow cytometry 48 hr after transfection. **D.** Immunoblot analysis (IB) of whole cell lysates (WCL) derived from B16 cells transfected with the indicated siRNA constructs. **E.** Numbers of survival B16 cells by knockdown of *Bcl-2* or/and *p53*. Equal numbers of B16 cells were transfected with vector or siRNA as indicated. The cells per well were determined under the microscope. Asterisks indicate statistical significance (**P < 0.05).

Our *in vivo* studies demonstrated that loss of *p53* inhibited the *Bcl-2*-null-induced melanocyte death (Figure 3). To identify whether this melanocyte lineage specific interaction between *p53* and *Bcl-2* is detectable *in vitro*, we silenced the expression of *Bcl-2* in mouse primary melanocytes isolated from wild-type *p53* mice or *p53*-null mice and then stained with toluidine blue to observe cell growth (Figure 5A) and counted cell numbers. Specifically, *Bcl-2* siRNA was transfected into wild-type and *p53*^−/−^ primary melanocytes. Cell number was counted at 48 hours after transfection. As shown in Figure 5B, more than half of the wild-type primary melanocytes died in comparison to the death of 20% of *p53*^−/−^ primary melanocytes (Figure 5B and 5C). This result indicates that the majority of *Bcl-2*-induced apoptosis is regulated by the optional *p53 in vitro*.

*Bcl-2*-targeted therapeutics are being widely explored [37]. To identify the dependence of *p53* status in *Bcl-2* targeted therapeutics in melanoma, we silenced the expression of *p53*, *Bcl-2* or both in B16 melanoma cells using siRNA approaches (Figure 5D) and then counted the cell numbers at 48 hours after silencing. In these experiments, Mitf expression was monitored to evaluate the silencing effects of *Bcl-2* [38]. We found that silencing *Bcl-2* inhibited B16 melanoma cell growth, especially in p53-absent cells (Figure 5E).

## DISCUSSION

In this study, we deleted the *p53* gene in *Bcl-2*^−/−^ mice to investigate the genetic interaction between *p53* and *Bcl-2 in vivo*. We found the *Bcl-2* knockout phenotype was partially alleviated by the deficiency of *p53*. Previous reports demonstrated *p53* can either induce the expression of pro-apoptotic *Bcl-2* proteins, or directly regulate a variety of *Bcl-2* proteins in the cytoplasm [39]. In the current study, we found that loss of *p53* rescued melanocyte stem cells from cell death and reduced hyper-apoptosis of spleenocytes in the *Bcl-2^−/−^* mouse. These results are consistent with previous reports that the absence of *p53* resulted in a broader resistance to death stimuli *in vivo* [39]. However, the absence of *p53* could not prevent *Bcl-2* deficient mice from polycystic kidney disease and thymus degeneration, indicating that *Bcl-2* deficiency-induced phenotype is *p53*-independent in kidney and thymus. Interestingly, previous reports showed that the *p53* response to γ-irradiation *in vivo* was also in a tissue-specific manner. Thus, tissue specific role of *p53* in apoptosis may be a generable phenotype [40]. Previous reports have demonstrated that loss of Bim rescue all defect in *Bcl-2*-null mice and indicate that Bim levels set the threshold for initiation of apoptosis [27]. Thus, our results also demonstrate the different role of *p53* protein and Bim protein in apoptosis. Our results further confirmed the gene-gene interaction is tissue specific. Multiple molecular mechanisms might involve in these tissue specific interactions. First of all, the expression levels of *p53* are different in different tissues. For example, we found that the expression of *p53* protein in mouse kidney is lower than in spleen, skin and thymus (Figure 2I). We further found that the interactions between *p53* protein and *Bcl-2* protein are different in kidney, spleen, skin and thymus (Figure 1, 2, 3 and 4). Second, co-factors in a protein complex are usually tissue specific. Other mechanisms, such as different transcriptional regulations in different tissues and different microRNAs expressions in different tissues, may also involve in the *Bcl-2/p53* interactions, all of which are deserved to be further studied. Further experiments are required to provide the detailed genetic and biochemical mechanisms for the tissue specific interaction between *p53* and *Bcl-2* in the regulation of cellular apoptosis.

Our *in vitro* data suggest that the majority of *Bcl-2*-induced apoptosis is regulated by the optional *p53* (Figure 5). This finding may encourage us to evaluate the therapeutic potential by the induction of apoptosis with *p53*- and *Bcl-2*-targeting in a tissue specific manner. Furthermore, our results preliminary suggest that *Bcl-2*-targeted therapeutics is more effective in melanomas with wild-type *p53* than in melanomas with mutant *p53* (Figure 5E). Due to loss of *p53* function, through *p53* mutation itself or perturbations in *p53* signaling pathways, is a common feature in the majority of human cancers. Our results indicate that *p53* status should be detected before *Bcl-2*-targeted therapeutics, which is suitable to be used in tumors with wild-type *p53*.

## MATERIALS AND METHODS

### Animals

*p53*-deficient (−/−) mice were C57BL/6 TSG-p53^®^ N12 and purchased from Taconic Farms (Hudson, NY, USA). These p53-deficient mice were originally generated by Donehower et al. [41]. *Bcl-2^−/+^* (C57BL/6J) mice were purchased from the Jackson laboratory. *DCT-lacZ* transgenic mice were a gift from I. Jackson. The *DCT-lacZ* transgenic colony (CBA/C57BL6) was backcrossed to C57BL/6J [26].

### Skin and hair growth assays

Dorsal skin sections were fixed, embedded in paraffin and stained with hematoxylin and eosin. For the hair growth assay, age-matched *p53*^+/+^, *p53*^−/−^, *Bcl-2^−/+^*, *Bcl-2^−/−^*/p53*^−/−^* and *Bcl-2^−/−^* mice (18 days) were shaved in a 2cm square area on their back near the tail using an electric razor and then depilated by Nair^®^. The subjective element in estimating the area is minimized by defining regrowth as the first appearance of hair, and dividing the shaved area to be scored into eight equal portions with a transparent screen [34, 35]. We measured hair regrowth 24 days after depilation.

### Whole mount β–galactosidase staining

Skin samples from *DCT-lacZ*, *bcl-2^−/−^*/DCT-lacZ**, *p53^−/−^*/DCT-lacZ**, and *p53^−/−^*/bcl-2*^−/−^*/DCT-lacZ** mice were stained as previously described [26]. Briefly, skin was fixed in X-Gal buffer (5 mM K_3_Fe (CN)_8_ postassium ferricyanide, 2 mM MgCl_2,_ 0.01% DOC, 0.02% NP40 and 0.05% X-Gal in PBS), incubated at 37° for 6 hours and cut by hand under a dissecting microscope.

### Histology

Animals were sacrificed by CO_2_ inhalation. Sections were immediately placed in 10% buffered formalin until paraffin embedding and sectioning (done by the Rodent Histopathology core service at Harvard Medical School). Hematoxylin/Eosin staining was performed by the histopathology core. Immunohistochemical studies were performed on skin specimens using formalin-fixed, paraffin-embedded tissue. Tissues known to express the antigen of interest were used as positive controls, whereas removal of the primary antibodies in the test tissues was used as negative controls. TUNEL kit was purchased from *R & D* and applied according to the manufacturer's instructions.

### siRNA

siRNA was from Dharmacon (Lafayette, CO). siControl contains four mismatches with all known human, mouse, and rat genes. Primary murine melanocytes were plated at <50% confluence (300,000 cells per 60-mm plate) 16–20 h before transfection in penicillin/streptomycin-free medium at a ratio of 6:1 (vol:vol) Oligofectamine (Invitrogen, Carlsbad, CA) to siRNA and incubated for 5–6 h per the manufacturer's instructions.

### Flow cytometry

Cells were fixed in ice-cold 70% ethanol before DNA staining with 50 μg/ml propidium iodide (Sigma Aidrich) in phosphate buffer saline containing 0.5 mg/ml RNase A (Amersha, inc. DNA content was analyzed by flow cytometry (Becton Dickinson FACSCalibur) [42].

### Cell growth assay

Cells were plated in 6-well plates and then transfected. 48 h after transfection cells were fixed in cold 100% methanol and cell growth was visualized by staining cells with 0.1% toluidine blue solution followed by destaining with 1% acetic acid. Images were documented with a FluorChem Imaging System (Bio-Rad).

## Abbreviations

MSC: melanocyte stem cell
PKD: polycystic kidney disease
CTL: cytotoxic lymphocyte
NK: natural killer
Palb2: Partner And Localizer of BRCA2
DCT: dopachrome tautomerase
WT: wild-type
UV: ultraviolet
DNA: deoxyribonucleic acid
Mitf: microphthalmia-associated transcription factor
Bg: bulge region
Bb: bulb
LE: lower enlargement
HE: Hematoxylin and eosin
siRNA: small interfering RNA
